# The Effects of Scanning Speed and Standoff Distance of the Fiber on Dusting Efficiency during Short Pulse Holmium: YAG Laser Lithotripsy

**DOI:** 10.3390/jcm11175048

**Published:** 2022-08-28

**Authors:** Junqin Chen, Daiwei Li, Wenjun Yu, Zhiteng Ma, Chenhang Li, Gaoming Xiang, Yuan Wu, Junjie Yao, Pei Zhong

**Affiliations:** 1 Thomas Lord Department of Mechanical Engineering and Materials Science, Duke University, Durham, NC 27708, USA; 2 Photoacoustic Imaging Lab, Department of Biomedical Engineering, Duke University, Durham, NC 27708, USA; 3 Department of Biostatistics and Bioinformatics, Duke University Medical Center, Durham, NC 27708, USA

**Keywords:** laser lithotripsy, fiber scanning speed, cavitation, mechanisms of stone dusting

## Abstract

To investigate the effects of fiber lateral scanning speed across the stone surface (*v_fiber_*) and fiber standoff distance (SD) on dusting efficiency during short pulse holmium (Ho): YAG laser lithotripsy (LL), pre-soaked BegoStone samples were treated in water using 0.2 J/20 Hz at SD of 0.10~0.50 mm with *v_fiber_* in the range of 0~10 mm/s. Bubble dynamics, pressure transients, and stone damage were analyzed. To differentiate photothermal ablation vs. cavitation damage, experiments were repeated in air, or in water with the fiber tip at 0.25 mm proximity from the ureteroscope end to mitigate cavitation damage. At SD = 0.10 mm, the maximum dusting efficiency was produced at *v_fiber_* = 3.5 mm/s, resulting in long (17.5 mm), shallow (0.15 mm), and narrow (0.4 mm) troughs. In contrast, at SD = 0.50 mm, the maximum efficiency was produced at *v_fiber_* = 0.5 mm/s, with much shorter (2.5 mm), yet deeper (0.35 mm) and wider (1.4 mm), troughs. With the ureteroscope end near the fiber tip, stone damage was significantly reduced in water compared to those produced without the ureteroscope. Under clinically relevant *v_fiber_* (1~3 mm/s), dusting at SD = 0.5 mm that promotes cavitation damage may leverage the higher frequency of the laser (e.g., 40 to 120 Hz) and, thus, significantly reduces the procedure time, compared to at SD = 0.1 mm that promotes photothermal ablation. Dusting efficiency during short pulse Ho: YAG LL may be substantially improved by utilizing an optimal combination of *v_fiber_*, SD, and frequency.

## 1. Introduction

Ureteroscopy with holmium: YAG (Ho: YAG) laser lithotripsy (LL) has become the first-line therapy for renal calculi over the past decade [1,2]. Driven by the development of high-power and high-frequency Ho: YAG lasers, stone dusting has gained clinical popularity over fragmenting because of the shortened procedure time and reduced risk of ureteral damage [3,4,5,6]. In dusting, the Ho: YAG laser is operated at a low pulse energy (Ep = 0.2–0.5 J) and a high pulse repetition rate or frequency (F = 12–100 Hz). By scanning the laser fiber at various speeds to “paint” over the stone surface line by line and layer by layer, dust-like fragments can be produced that may be discharged spontaneously without the need for basket extraction [5,7,8]. The dusting procedure, however, is carried out empirically based on urologist’s experience with no consensus on the optimal settings, such as fiber tip-to-stone standoff distance (SD) and fiber scanning speed (*v_fiber_*). In addition, the mechanisms of stone dusting produced by a moving fiber during LL remains to be elucidated.

Conventional theory attributes photothermal ablation and microexplosion to be the dominant mechanisms of stone damage in fragmenting during LL [2,9,10], primarily based on studies using high *E_p_* (≥0.8 J), low F (<10 Hz), and long pulse durations (>100 μs). As a result, placing the fiber tip in contact with the stone surface, i.e., SD = 0 mm is often advocated, with the hope to maximize laser energy transmission and treatment efficiency [11,12,13,14]. Clinically, however, maintaining a close contact of the fiber tip with the stone surface is challenging during LL. In particular, a moving fiber cannot be placed precisely in constant contact with the stone due to either changing surface curvature of the stone [13], the retropulsion effect [15,16], or respiration-induced kidney movement [5]. Moreover, a panel of leading endourologists have recently recommended that dusting efficiency can be improved clinically by “defocusing” the laser beam via pulling back the fiber tip slightly away from the stone surface to produce small fragments [5], suggesting that a different mechanism of stone damage may emerge during dusting with low *E_p_* (<0.5 J), high F (≥20 Hz), and short pulse durations (<100 μs).

Physically, the laser pulse energy deliverable to the stone will decrease significantly even at a short SD (<1 mm) because of the shallow optical penetration depth (~0.4 mm) of the Ho: YAG laser in water [17,18]. This characteristic of the Ho: YAG laser, coupled with the low *E_p_* used for stone dusting, may diminish the contribution of photothermal ablation [19]. Consequently, most of the laser energy in the dusting procedures is likely absorbed by the interposing fluid between the fiber tip and the remaining stone surface, leading to the formation of a cavitation bubble with subsequent expansion and collapse [19,20], and temperature rise inside the urinary tract [21,22]. Using a short pulse (70 to 78 μs) Ho: YAG laser, we have recently demonstrated that cavitation bubble collapse plays a critical role in stone dusting using a stationary fiber (365 mm core diameter), the efficiency of which can be substantially improved at an optimal SD of 0.5 mm [19].

Several groups have investigated the effect of fiber scanning on stone damage *in vitro* during Ho: YAG LL, mostly in contact mode (i.e., SD ≈ 0 mm where photothermal effects dominate). At *E_p_* = 0.5 J and F = 20 Hz (with a 230 mm core diameter), Alhoukhi and colleagues compared two clinically relevant fiber scanning speeds and found that the mass loss was much greater at 3 mm/s than at 1 mm/s [13]. Using similar settings (with a 272 mm core diameter), Panthier et al. demonstrated that the maximum stone damage could be produced at *v_fiber_* = 10 mm/s or 2.5 mm/s, compared to lower scanning speeds [23]. Both studies used BegoStone phantoms, yet the mechanism that led to maximum efficiency in stone dusting was not investigated. Moreover, two distinctly different damage patterns (i.e., long and continuous trough vs. discrete craters) were produced by either increasing *v_fiber_* from 8.3 mm/s to 25 mm/s [24] or by decreasing the number of pulses delivered per scanning distance from 10 pulses/mm to 1 pulse/mm [25]. These previous observations suggest that stone damage during scanning treatment may correlate with the overlapping area ratio (OAR) between successive laser pulses, which effectively combines the contribution of *v_fiber_*, F, and fiber size together in stone dusting.

In this work, we investigate the effect of *v_fiber_* at three different SDs (0.10, 0.25, and 0.50 mm) under clinically relevant settings for short pulse Ho: YAG LL. At F = 20 Hz, *v_fiber_* was varied from 0 to 10 mm/s so that a full range of OAR from 0% to 100% could be achieved during scanning, which were correlated with the resultant stone damage. Moreover, the contribution of photothermal ablation vs. cavitation to stone dusting was differentiated by comparing damage troughs produced in water vs. in air, and with vs. without the use of a ureteroscope that alters the direction of bubble collapse in water. Overall, our results demonstrate that cavitation plays an indispensable role in stone dusting under various SDs during short pulse Ho: YAG LL. The treatment efficiency may be significantly improved, with the procedure time greatly shortened, by an optimal combination of *v_fiber_*, SD, and F.

## 2. Materials and Methods

### 2.1. Sample Preparation Protocol

BegoStone samples (BEGO USA, Lincoln, RI, USA) with similar mechanical properties to human kidney stones [26,27] were prepared using a 5:2 powder to water ratio by weight [28]. Briefly, the mixture was poured into a mold (23 mm × 23 mm × 10 mm, L × W × H) and agitated on an orbital shaker (KS 130 control, IKA Works, Wilmington, NC, USA) at 600 RPM for 15 min, which localized residual air pockets and impurities in the central region of the sample [28]. After a 24 h curing period, the stone samples were removed from the mold and the two flat surfaces were polished using 1200-grit sandpaper until the sample height was reduced to 4 mm while visible voids were removed from the surface. This preparation procedure allowed us to produce uniform sample surfaces, which improves the consistency of the experimental results. All stone samples were soaked in water for 24 h before LL treatment.

### 2.2. Scanning Fiber Experiments

**Figure 1:**
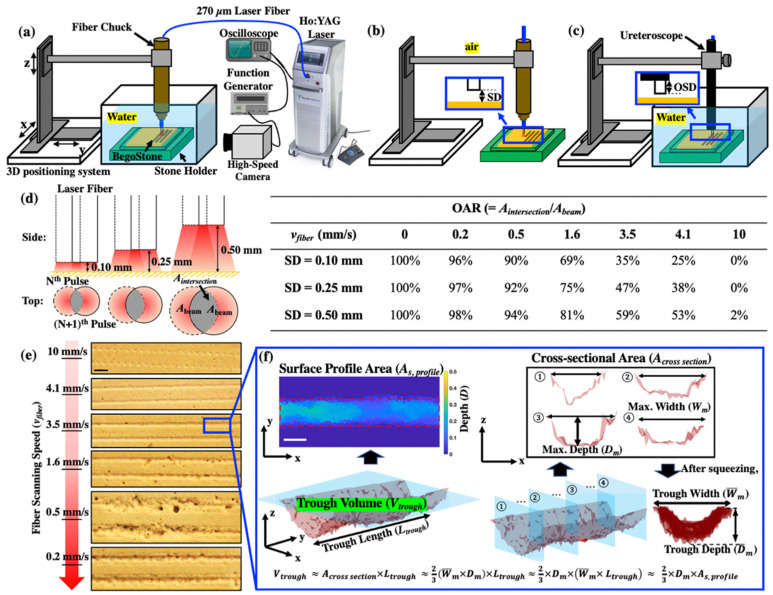
Experimental setup for pre-soaked BegoStone samples treated with perpendicular fiber (core diameter = 270 mm) placed at different fiber tip-to-stone standoff distances (SDs) (**a**) in water, synced with high-speed imaging from the side-view, (**b**) in air, and (**c**) in water with a ureteroscope placed at various offset distances (OSDs). A closer view of the gaps between the scope end, fiber tip, and stone surface are shown in the blue boxes. (**d**) Overlapping area ratio (OAR) at different SDs and *v_fiber_*, which was calculated by Equation (2). (**e**) Representative images of stone damage produced at different *v_fiber_* (scale bar = 1 mm). (**f**) An example of a damage trough, which was 3D reconstructed and quantified by OCT scanning, including trough volume, surface profile area, trough width, and depth (scale bar = 0.5 mm).

Pre-soaked BegoStone phantoms were treated in water at *E_p_* = 0.2 J and F = 20 Hz for dusting using a Ho: YAG laser lithotripter (H Solvo 35-watt laser, Dornier MedTech, Munich, Germany) with a full width at half maximum (FWHM) pulse duration of 70 μs (Figure 1a). A small 270 μm core diameter fiber (Dornier SingleFlex 200, NA = 0.26, Munich, Germany) was chosen because of its compatibility with flexible ureteroscopy [29,30]. The stones were fixed in a water tank filled with degassed water at room temperature. The laser fiber held by a fiber chuck was positioned perpendicularly to the stone surface, i.e., at a 0° laser incident angle, using a 3D positioning stage (VXM-2 step motors with BiSlide-M02 lead screws; Velmex, Bloomfield, NY, USA).

A custom MATLAB program (Math-Works, Natick, MA, USA) was used to precisely control the initial SD (=0.10, 0.25 and 0.50 mm) of the fiber tip to the stone surface during fiber scanning at each selected *v_fiber_* [14,25]. Lines of damage troughs were created (see Figure 1e). Following the scan of lines about 20 mm long, a new fiber tip was prepared, as needed, using a fiber stripper, and cleaved using ceramic scissors. The fiber tip condition was confirmed by inspecting the quality of the laser aiming beam on a flat white surface [25,28].

### 2.3. Quantitative Analysis of Stone Damage

Post-LL, the damage troughs on the stone surface produced at 0 < *v_fiiber_* < 10 mm/s were quantified by optical coherence tomography (OCT, OQ Labscope, Lumedica, Durham, NC, USA). The total length of the trough (*L_trough_*) produced by 100 pulses was measured, which is proportional to the product of *v_fiber_* and treatment time. Since the maximum dimension quantifiable by our OCT device was 7 mm × 7 mm × 2.5 mm (L x W × H), for damage produced at *v_fiber_* > 1 mm/s, we scanned multiple troughs of equal lengths and summed up the results to obtain the total trough volume (*V_trough_*), which was used to assess dusting efficiency under each test condition. The fiber speed that led to the maximum dusting efficiency is considered as the optimal *v_fiber_* for the settings used in this work. In addition, assuming that uniform damage troughs were produced by the Gaussian profile of the laser beam [31] with a mean parabolic cross-sectional area (${\bar{A}}_{cross\;section}$), *V_trough_* could be estimated by the following equation:

(1) $$V_{trough}\approx {\bar{A}}_{cross\;section}\times L_{trough}\approx \frac{2}{3}\left({\bar{W}}_{m}\times {\bar{D}}_{m}\right)\times L_{trough}\approx \frac{2}{3}\times {\bar{D}}_{m}\times A_{s,\;profile},$$

where ${\bar{W}}_{m}$ and ${\bar{D}}_{m}$ are the means of the maximum width and depth of the cross sections, respectively, and $A_{s,profile}$ (=${\bar{W}}_{m}\times L_{trough}$) is the surface profile area of the trough. A custom program developed in MATLAB [28] was used to extract these parameters from each damage trough based on the acquired OCT images (see Figure 1f).

In particular, for the single damage craters produced at *v_fiber_* = 0 mm/s, the maximum width along the direction of the fiber movement (*y*-axis) was used as $L_{trough}$, while the mean of the maximum width of the cross sections (along *x*-axis) was taken as ${\bar{W}}_{m}$. For the individual craters produced at *v_fiber_* = 10 mm/s, we used the average of ${\bar{W}}_{m}$ for each individual crater to represent the final ${\bar{W}}_{m}$ and the summation of the $L_{trough}$ from individual craters to represent the total $L_{trough}$ under the test condition.

### 2.4. Assessment of Different Damage Mechanisms

Stone damage in LL could be produced by different mechanisms [2,9,10,19,20,28]. To eliminate cavitation-induced stone damage, we performed a second set of experiments in air, which maximizes the laser energy transmission to the stone surface and photothermal ablation [9,28]. In addition, we carried out a third set of experiments in water by advancing the fiber tip beyond the distal end of a flexible ureteroscope (Dornier AXIS^TM^, 3.6 F working channel, Munich, Germany) with a short offset distance (OSD) of 0.25 mm. This method was used to mitigate the bubble collapse toward the stone boundary and, thus, minimize cavitation-induced damage without affecting the MOSES effect and photothermal ablation of the stone material by the laser pulses in water [19,20,32]. Using these strategies, we could differentiate the contribution of photothermal ablation vs. cavitation damage in stone dusting. For completeness, we also evaluated the effect of the ureteroscope on stone dusting efficiency at two clinically relevant OSDs of 2 mm and 3 mm [33], under the optimal *v_fiber_* at different SDs.

### 2.5. Effect of the Overlapping Area Ratio (OAR) on Dusting Efficiency

Stone damage produced by a scanning fiber during LL may depend on the overlap of laser pulses in the irradiated region [24,34]. We calculate OAR between two successive laser pulses for a constant *v_fiber_* by using the following equation [35]:

(2) $$OAR=\frac{A_{intersection}}{A_{beam}}=\frac{2\left[{\left(r_{beam}\right)}^{2}\cos^{-1}\left(\frac{v_{fiber}}{2F\;r_{beam}}\right)-\frac{v_{fiber}}{2F}\sqrt{{\left(r_{beam}\right)}^{2}-{\left(\frac{v_{fiber}}{2F}\right)}^{2}}\right]}{\pi \;{\left(r_{beam}\right)}^{2}}$$

where *A_intersection_* is the intersection area between two laser beams in water (see Figure 1d), *A_beam_* is the projected beam area on the stone surface, $r_{beam}$ is the projected beam radius (0.16 mm, 0.20 mm and 0.27 mm at SD = 0.1, 0.25 and 0.5 mm, respectively), and $\frac{v_{fiber}}{F}$ is the inter-pulse distance traveled by the scanning fiber [34]. As OAR approaches 0%, there is no overlap between subsequent pulses, leading to the formation of individual shallow craters on the stone surface (see Figure 1e at 10 mm/s). In comparison, at an OAR of 100%, the laser pulses are fully overlapped with each other, leading to the deep and saturated craters, such as those produced by a stationary fiber of a 365 μm core diameter fiber under the same *E_p_* and F in our previous study [19].

### 2.6. High-Speed Imaging and Pressure Transient of Bubble Dynamics

To capture the bubble dynamics produced in water, we fixed the fiber in position and translated the stone at the selected *v_fiber_* in the opposite direction (i.e., *v_stone_* = −*v_fiber_*). A digital time delay generator (BNC 565, Berkeley Nucleonics Corporation, San Rafael, CA, USA) was used to trigger a Kirana5M high-speed video camera (Specialised Imaging, Pitstone, UK) operated at 200,000 frames per second using the internal photodetector signal from the laser [28]. Moreover, the bubble-induced pressure transients were measured by a needle hydrophone (HNC-1000, Onda, Sunnyvale, CA, USA) at 45° angles, placed at about 30 mm from the fiber tip. Furthermore, the experiment in air was recorded to visualize the material ejection from the wet stone surface, using a Phantom v7.3 high-speed video camera (Vision Research, Wayne, NJ, USA) operated at 90,909 frames per second.

### 2.7. Statistical Analysis

Three-way ANOVA analyses were first carried out to assess the contribution of three factors—treatment condition (in air or in water), *v_fiber_*, and SD (0.10 mm, 0.25 mm, and 0.50 mm)—on stone dusting efficiency based on the F-test. If any of these F-tests is significant (*p* < 0.05), we then performed a post-hoc test, the Tukey’s honestly significant difference (TukeyHSD) test to determine which groups were different from one another. Based on the TukeyHSD test results, we could identify the optimal *v_fiber_* and SD among the various combinations of the test conditions. In addition, we have used two-sample *t*-tests to compare stone dusting efficiency produced at different OSD levels associated with the flexible ureteroscope.

## 3. Results

### 3.1. Stone Damage Produced in Water under Different v_fiber_ and SDs

**Figure 2:**
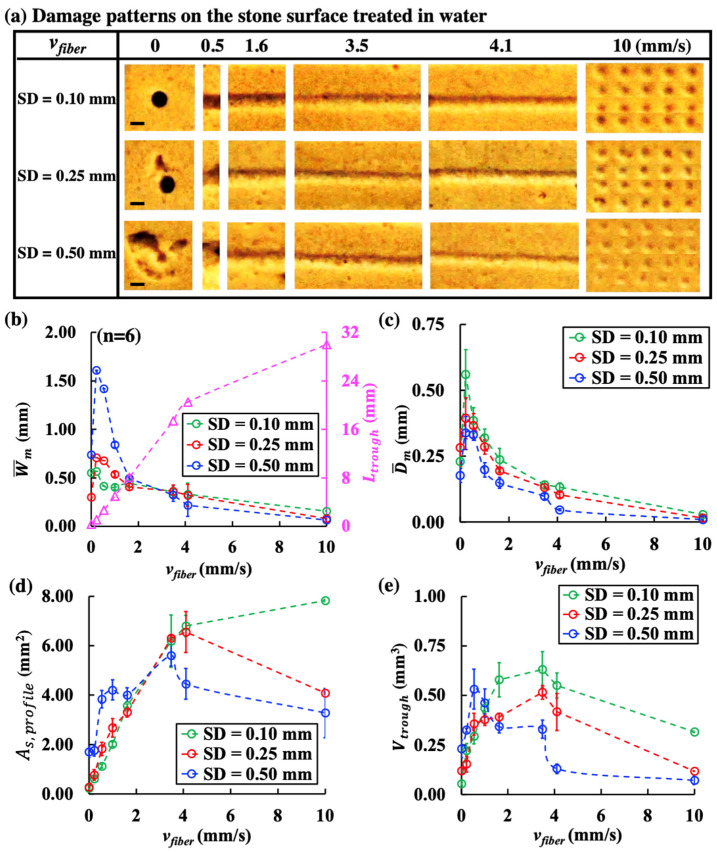
Different damage patterns and characteristic dimensions of the trough produced in water by 100 pulses (0.2 J and 20 Hz) during scanning treatment at various fiber speeds (*v_fiber_*). (**a**) Damage craters produced by the stationary fiber (*v_fiber_* = 0 mm/s) and one-fifth of the damage troughs created at different *v_fiber_* under SD = 0.10, 0.25, and 0.50 mm (scale bar = 0.5 mm), (**b**) mean trough width (${\bar{W}}_{m}$) (small circles in different colors) and total trough length (*L_trough_*) (small purple triangles), (**c**) mean trough depth (${\bar{D}}_{m}$), (**d**) surface profile area (*A_s, profile_*), and (**e**) trough volume (*V_trough_*) quantified by OCT imaging analysis and plotted vs. *v_fiber_*.

Figure 2a shows the damage patterns produced under different *v_fiber_* at various SDs in water. Several important features could be observed. First, for a stationary fiber (i.e., *v_fiber_* = 0 mm/s), a small circular crater was formed at SD = 0.10 mm, while irregularly shaped craters with enlarged surface profile areas were produced at SD = 0.25 and 0.50 mm. The additional damage around the central crater might be produced by the toroidal bubble collapse following the primary collapse of the LL-induced vapor bubble [19]. Second, for a scanning fiber, straight damage troughs with varying degrees of *L_trough_*, ${\bar{W}}_{m}$, and ${\bar{D}}_{m}$ were produced. Notably, when *L_trough_* lengthened significantly at increased *v_fiber_*, the corresponding values of ${\bar{W}}_{m}$ and ${\bar{D}}_{m}$ would decrease appreciably in the range of *v_fiber_* = 0.5–10 mm/s (Figure 2b,c). Interestingly, the ${\bar{W}}_{m}$ and ${\bar{D}}_{m}$ of the craters produced at 0 mm/s were also smaller than their counterparts produced at 0.5 mm/s. Third, at *v_fiber_* = 10 mm/s, individual and shallow circular craters with uniform spacing between them were produced since there was no overlap between two successive fiber irradiation positions during scanning. Under such high *v_fiber_*, the ${\bar{W}}_{m}$, ${\bar{D}}_{m}$ and $A_{s,profile}$ (Figure 2b–d) of the individual craters would all decrease with increasing SD.

More importantly, both *v_fiber_* and SD were found to exert statistically significant influence on *V_trough_* (*p* < 0.001) with the optimal *v_fiber_* for maximum dusting efficiency varying distinctly with SDs (Figure 2e). At SD = 0.10 mm and SD = 0.25 mm, the trough volumes initially increased with *v_fiber_* and reached their peak values of 0.63 mm^3^ and 0.51 mm^3^, respectively, at *v_fiber_* = 3.5 mm/s, before decreasing gradually from *v_fiber_* = 4.1 mm/s to 10 mm/s. In comparison, at SD = 0.50 mm, the maximum trough volume of 0.53 mm^3^ was produced at an optimal *v_fiber_* = 0.5 mm/s before tapering off thereafter.

### 3.2. Contribution of Photothermal Ablation vs. Cavitation to Stone Dusting

To differentiate the mechanism of stone dusting under various treatment conditions (*v_fiber_* and SD), we compare stone damage produced in water with or without the flexible ureteroscope (or scope for brevity henceforth) at OSD = 0.25 mm, as well as in air.

#### 3.2.1. Different Bubble Dynamics and Stone Damage Characteristics Produced by Photothermal Ablation and Cavitation Bubble Collapse

**Figure 3:**
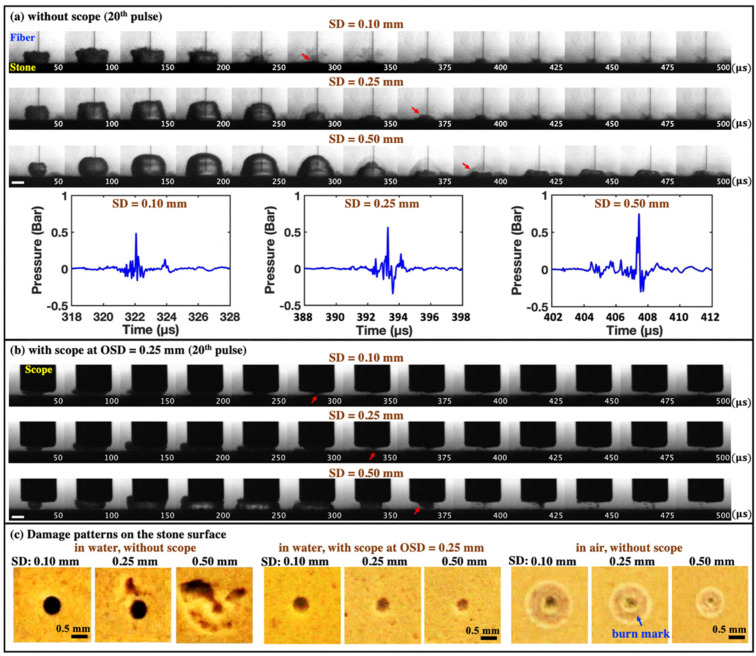
Representative high-speed imaging sequences of bubble dynamics produced in water near the BegoStone surfaces during dusting (*E_p_* = 0.2 J and F = 20 Hz) using a stationary fiber. (**a**) Without the ureteroscope (or scope) at SD = 0.10 mm, 0.25 mm, and 0.50 mm and the corresponding pressure transients measured by the needle hydrophone, and (**b**) with the scope at OSD = 0.25 mm. In both (**a**,**b**), the red arrows indicate the direction of bubble collapse under different treatment conditions, and the scale bar = 1 mm. (**c**) Damage patterns produced on the BegoStone surfaces after 100 pulses in water without and with the scope, and in air. The blue arrow indicates the burn mark around the central craters.

Figure 3 shows representative high-speed images of the bubble dynamics produced by the laser–fluid–stone interaction at different SDs using a stationary fiber. Without the scope, the maximum bubble size formed was found to increase with SD from 0.10 mm to 0.50 mm as more laser pulse energy was absorbed by the interposing fluid between the fiber tip and stone surface, as observed previously [19]. Consequently, the strongest pressure transient was generated by the bubble collapse at SD = 0.50 mm (Figure 3a). With the scope at OSD = 0.25 mm, although the bubble expansion in relation to laser transmission to the stone (i.e., the MOSES effect) were not affected, the collapse of the bubble was distracted by the proximity of the scope tip and moved away from the stone surface (Figure 3b). When the bubble collapse was mitigated by the scope tip, the resultant craters became much smaller and shallower, compared to their counterparts produced without the scope at different SDs (Figure 3c). Furthermore, stone damage produced in air without cavitation was similar to the small circular craters produced in water with the scope, except that the sizes of the central crater and surrounding burn mark would decrease with increasing SD (Figure 3c).

#### 3.2.2. Cavitation Bubble Collapse toward the Stone Surface Is Indispensable in Stone Dusting

**Figure 4:**
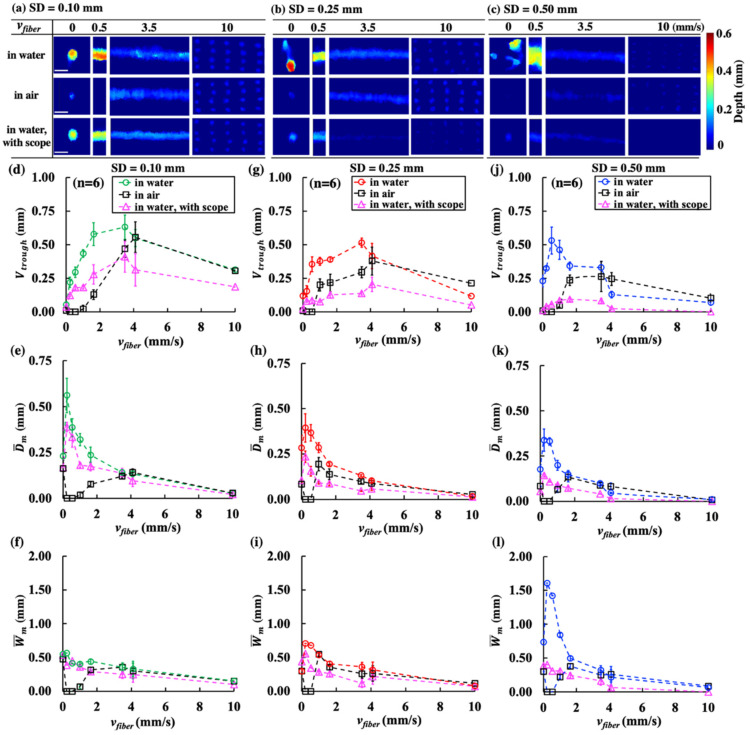
OCT reconstruction of damage patterns on BegoStone surfaces produced by 100 pulses (0.2 J and 20 Hz) in water and in air without scope, and in water with scope at OSD = 0.25 mm delivered at (**a**) SD = 0.10 mm, (**b**) SD = 0.25 mm, and (**c**) SD = 0.50 mm (scale bar = 1 mm). Trough volume (*V_trough_*) (**d**,**g**,**j**), mean trough depth (${\bar{D}}_{m}$) (**e**,**h**,**k**), and mean trough width (${\bar{W}}_{m}$) (**f**,**i**,**l**) are plotted vs. *v_fiber_*.

In general, *V_trough_* produced in water without the scope is significantly larger than its counterpart with the scope (Figure 4), confirming the indispensable role of cavitation bubble collapse in stone dusting [19] even during scanning treatment. Although the variations of damage patterns with *v_fiber_* at different SDs were similar, the largest reduction in stone damage by the proximity of the scope tip was 5.3-fold, observed at SD = 0.50 mm under the optimal *v_fiber_* of 0.5 mm/s. In comparison, the maximum reduction in stone damage at SD = 0.10 mm and SD = 0.25 mm were 1.5- and 2.5-fold, respectively, observed under a different optimal *v_fiber_* of 3.5 mm/s. These significant reductions in *V_trough_* may be attributed to the substantial diminishments in both ${\bar{D}}_{m}$ and ${\bar{W}}_{m}$ because of the suppressed bubble collapse, especially for SD = 0.50 mm (Figure 4e–l).

Even without cavitation and with more laser energy delivered to the stone in air [9,10,28], the resultant stone damage was still found to be less than its counterpart produced in water without the scope for *v_fiber_* < 4.1 mm/s, regardless of SD. These findings confirm again that besides photothermal ablation, cavitation contributes vitally to stone dusting in short pulse Ho: YAG LL [19].

#### 3.2.3. Effects of *v_fiber_* and SD on Bubble Dynamics and Resultant Acoustic Emission

**Figure 5:**
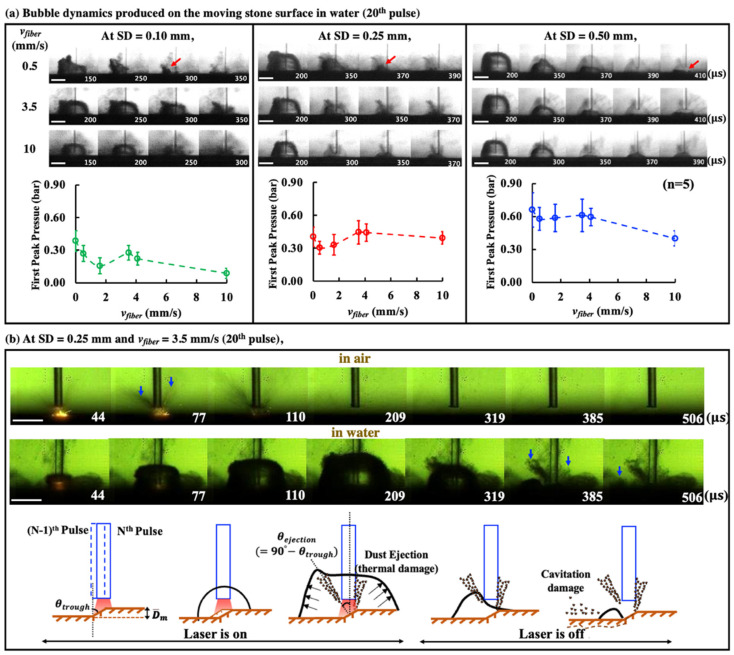
(**a**) High-speed imaging sequences in which the bubble produced on the stone surface moving at different speeds (*v_fiber_*) expands to its maximum volume and collapses at different SDs (scale bar = 1 mm). The red arrows indicate the direction of bubble collapse. The first peak pressure obtained from the hydrophone measurements is plotted vs. *v_fiber_*. The direction of stone movement in the high-speed imaging was from right to left. (**b**) Representative high-speed imaging sequences of laser-stone interaction in air vs. in water at one of the optimal settings (SD = 0.25 mm, *v_fiber_* = 3.5 mm/s) (scale bar = 1 mm) and the sketch of general process of stone dusting produced in water, where $\theta_{trough}$ is the angle of trough damage produced by the first N pulse, and $\theta_{ejection}$ is the angle of material ejection from stone surface. The blue arrows indicate the material ejection.

Representative high-speed imaging frames of bubble expansion and collapse at different SDs and *v_fiber_* are shown in Figure 5a. To facilitate high-speed imaging, the camera and the fiber were fixed during the experiment, while the stone was translated at different speeds (from right to left) to mimic the scanning treatment. The shape of the bubble expansion was significantly altered by the movement of the stone, especially at short SD (e.g., 0.10 mm) and slow *v_fiber_* (e.g., 0.5 mm/s). At a low *v_fiber_* of 0.5 mm/s, the maximum bubble size increased appreciably compared to its counterpart at the high *v_fiber_* of 10 mm/s, regardless of SDs. This difference is likely caused by the more rapid growth of the damage trough produced at a slow scanning speed, leading to a greater ${\bar{W}}_{m}$ and ${\bar{D}}_{m}$ (Figure 4e–l) and, thus, increased laser absorption in the interposing fluid than those produced at a fast-scanning speed. Concomitantly, the highest acoustic pressure emitted by the bubble collapse was measured at SD = 0.50 mm, followed by SD = 0.25 mm, with the lowest acoustic emission produced at SD = 0.10 mm (Figure 5a). Furthermore, the peak pressure varied significantly with SD, and statistical differences were observed in the acoustic emission between *v_fiber_*_ ≤_ 4.1 mm/s and *v_fiber_* = 10 mm/s (*p* < 0.04). These results are consistent with the general notion that the effect of cavitation in stone dusting will increase with a slight SD, reaching a maximum at the optimal SD of 0.50 mm for short pulse Ho: YAG lasers (Figure 4).

Figure 5b shows an example of the detailed physical processes involved in air vs. in water during scanning treatment under one of the optimal settings (SD = 0.25 mm, *v_fiber_* = 3.5 mm/s). The laser–stone (in air) or laser–fluid–stone (in water) interaction during scanning treatment created by the N^th^ pulse was influenced by the surface damage already produced by the previous (N-1) pulses. As such, while a portion of the laser pulse energy irradiated (as the fiber moved equivalently from left to right) was absorbed by the trailing damage trough surface, the rest of the energy would be absorbed by the untreated surface in front of the scanning fiber (toward its right side). As a result, two distinctly different directions of material ejection could be observed in front and behind the scanning fiber (see arrows at 77 µs in Figure 5b). In general, previous studies [15,36] suggest that the central direction of material ejection caused by photothermal ablation is approximately perpendicular to the stone surface, which is consistent with our observations in air.

In comparison, the vapor bubble created in water during scanning was significantly distorted (especially at low speed and short SD, see Figure 5a) compared to the bubble produced by a stationary fiber (Figure 4a). The bubble dimension was larger behind (left) than in front of the fiber (right), presumably due to the stronger laser absorption in the interposing fluid in the damage trough formed behind the fiber, supplemented by the material ejection and laser-dust interaction (i.e., flashes inside the expanding bubble at 44 µs in Figure 5b). Following the cessation of the laser pulse and the maximum expansion, the bubble tended to collapse toward the left into the damage trough (see the sketch in Figure 5b). This asymmetric expansion and collapse of the bubble produced by a scanning fiber (see Video S1) may cause additional material removal by the multi-foci collapse of the bubble (see arrows at 385 µs and 506 µs in Figure 5b) following the initial photothermal damage.

#### 3.2.4. Effect of Fiber Tip OSD from the Ureteroscope on Stone Dusting Efficiency

**Figure 6:**
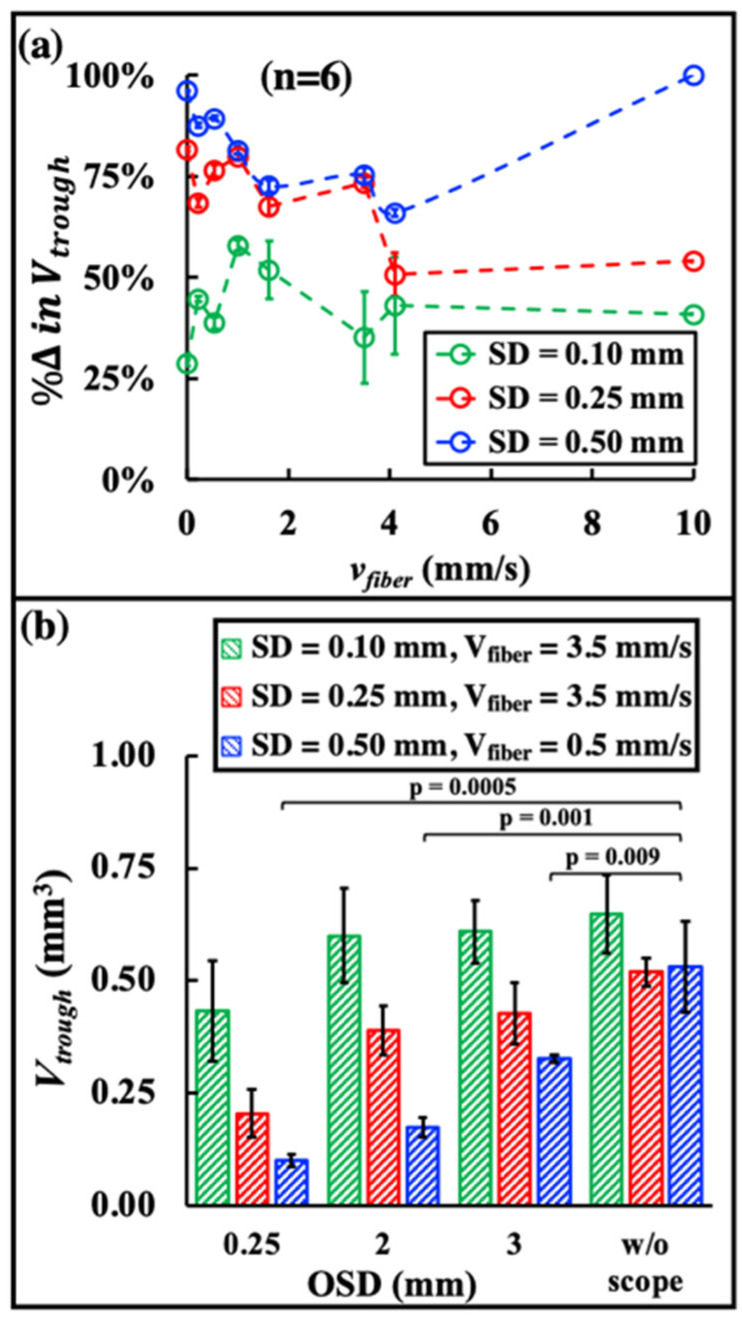
(**a**) The percentage of reduction in trough volume ($\%\Delta \;\mathrm{in}\;V_{trough}$) at different SDs and *v_fiber_* were calculated by $\%\Delta \;in\;V_{trough}$ = $\frac{V_{w/o\;scope}-V_{with\;scope}}{V_{w/o\;scope}}\times 100\%,$ where $V_{w/o\;scope}$ is the trough volume produced by the treatment without the scope and $V_{with\;scope}$ is the trough volume produced by the treatment with the scope at OSD = 0.25 mm. (**b**) Trough volume vs. OSDs under the three optimal conditions, as follows: SD = 0.10 mm at *v_fiber_* = 3.5 mm/s, SD = 0.25 mm at *v_fiber_* = 3.5 mm/s, and SD = 0.50 mm at *v_fiber_* = 0.5 mm/s.

When the bubble collapse to the stone surface was mitigated by the scope tip at OSD = 0.25 mm (Figure 4d–j), the resulting *V_trough_* was reduced by 29–58% for SD = 0.10 mm, 51–82% for SD = 0.25 mm, and 66–100% for SD = 0.50 mm in the range of *v_fiber_* = 0–10 mm/s (Figure 6a) compared to those produced without the scope. These results clearly demonstrate the critical role that cavitation bubble collapse plays in stone dusting, especially for SD = 0.25 and 0.50 mm at a slow scanning speed (i.e., *v_fiber_* ≤ 3.5 mm/s). Clinically, the OSD is typically in the range of 2–3 mm [33]. Under such conditions, the presence of the ureteroscope tip on stone damage was found to be statistically insignificant under the optimal speed *v_fiber_* = 3.5 mm/s for SD = 0.10 mm or 0.25 mm (*p* > 0.4) (Figure 6b). Only under SD = 0.50 mm with its optimal speed *v_fiber_* = 0.5 mm/s, was the reduction in *V_trough_* at OSD = 2 or 3 mm 67% or 38%, respectively, which was statistically significant (*p* < 0.01).

## 4. Discussion

The introduction of high-power (up to 120 W) and high-frequency (up to 120 Hz) Ho: YAG lasers in recent years has fundamentally changed the mode of LL from fragmenting to dusting, pop-dusting, or popcorning [5,8]. These new treatment modes significantly reduce the overall procedure time, eliminate the need for stent use while minimizing ureteral damage in LL [37,38]. Despite these advances, fundamental challenges still exist in LL to improve stone dusting efficiency via optimization of treatment strategy [39,40,41,42]. In this work, we have demonstrated that the efficiency of stone dusting could be maximized by an optimal combination of *v_fiber_* and SD produced by a short pulse Ho: YAG laser at 20 Hz, as discussed in further detail below.

The overarching goal in stone dusting is to create fine fragments (e.g., <1 mm) while maximizing the material removal [8,16]. Dusting efficiency may be influenced by multiple settings, and procedural parameters in LL, including *E_p_*, F, SD, *v_fiber_*, pulse profile, and sequence, in addition to stone composition, shape and size [16,43,44,45,46]. Clinically, it has been advocated that dusting should be performed using the lowest possible *E_p_* to create fine fragments while minimizing the undesirable retropulsion effect [16]. Moreover, careful adjustment of the fiber tip to stone surface distance (or SD) has been recommended for clinical LL [5], and its influence on treatment outcome demonstrated recently in laboratory studies [19,28]. Furthermore, scanning the fiber tip over the stone surface may avoid saturation in stone damage when laser pulses are delivered to a fixed spot [23,47]. However, the optimal *v_fiber_* and SD have not been determined, nor has the associated mechanism of action been elucidated. In this study, combining high-speed imaging and hydrophone measurement, with the proximity effect of the ureteroscope tip, we shed new light on the physical processes and critical parameters that may profoundly influence dusting efficiency during scanning treatment using short pulse Ho: YAG lasers.

**Figure 7:**
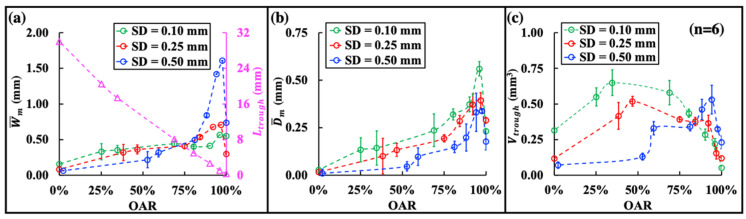
(**a**) Total trough length (*L_trough_*) (small purple triangles) and mean width (${\bar{W}}_{m}$) (small circles in different colors), (**b**) mean depth (${\bar{D}}_{m}$ ), and (**c**) volume (*V_trough_*) of the damage trough vs. the overlapping area ratio (OAR) between the successive laser pulses.

Our results suggest that maximum material removal during dusting is caused by the combined effects of the three characteristic dimensions in the damage trough (i.e., *L_trough_*, ${\bar{D}}_{m}$ and ${\bar{W}}_{m}$). Importantly, the optimal treatment conditions and associated mechanism of action depend critically on SD and the OAR between successive laser pulses (Figure 7). For example, in contact mode (i.e., SD = 0.10 mm) when photothermal ablation dominates, the maximum dusting efficiency is produced at an optimal *v_fiber_* of 3.5 mm/s, corresponding to an OAR of 35%. Under such treatment conditions, *V_trough_* will increase rapidly with *v_fiber_* and reach a maximum at *v_fiber_* = 3.5 mm/s before tapering off thereafter. This optimization process is driven primarily by the significant increase in *L_trough_* (see Figure 4a) before the concomitantly reduced ${\bar{D}}_{m}$ and ${\bar{W}}_{m}$ diminish markedly (see Figure 4e,f).

In contrast, in non-contact mode (e.g., SD = 0.50 mm) when cavitation-enhanced stone dusting could be significantly boosted using short pulsed Ho: YAG lasers [19] while the photothermal effect is substantially reduced, the maximum dusting efficiency is produced at a much slower optimal *v_fiber_* of 0.5 mm/s, corresponding to a significantly increased OAR of 94%. Under such treatment conditions, the significant overlap between successive laser pulses will greatly enhance the accumulative effect of cavitation damage [19] with substantially broadened ${\bar{W}}_{m}$ and deepened ${\bar{D}}_{m}$ (see Figure 4k,l), despite the fact that the increase in *L_trough_* is limited (see Figure 4c).

Although the significant contribution of cavitation to stone dusting has been recently demonstrated using fixed spot treatment with up to 640 pulses [19], there is concern regarding whether such a critical observation will hold true under clinically relevant LL conditions [41,42]. As such, it is important to note that when the contribution of bubble collapse was mitigated (Figure 6a), stone dusting efficiency could be greatly reduced (51% to 100%) during scanning at various *v_fiber_* from SD = 0.25 mm to 0.5 mm. Even at SD = 0.1 mm, when a strong photothermal effect was anticipated, a remarkable decrease in stone damage (29% to 58%) could still be observed by altering the bubble collapse.

Altogether, these results imply an indispensable role of cavitation in stone dusting from every single bubble collapse. As captured by the high-speed imaging in Figure 5, the bubble collapse may help removal of dust or damaged substances from the trough surface, and, thus, enhance the energy transmission of subsequent laser pulses to reach the underneath stone material for effective photothermal ablation. Such a scenario may present an alternative mechanism by which the collapse of cavitation bubbles and associated flow streaming, even under suboptimal conditions (i.e., contact mode or SD = 0.1 mm), may contribute to stone damage. In contrast, when using a non-contact mode at a SD conductive for cavitation damage (e.g., SD = 0.5 mm), the dynamics of bubble collapse will be critically influenced by the topology of the stone surface and proximity to the scope tip (Figure 5 and Figure 6b). Further studies are warranted to elucidate the mechanism of action for cavitation-driven stone dusting under clinically relevant treatment conditions and inside the kidney in tissue surrounded environment [42].

Moreover, it is worth noting when *v_fiber_* increases from 0.2 mm/s to 10 mm/s, the significant downward trends in ${\bar{D}}_{m}$ and ${\bar{W}}_{m}$ observed in Figure 4 are much more pronounced in water without the scope (i.e., strong bubble collapse) than in water with the scope at OSD = 0.25 mm (i.e., mitigated bubble collapse). Therefore, without bubble collapse, a higher *v_fiber_* is needed to produce a large *L_trough_* that compensates the reduction in ${\bar{D}}_{m}$ and ${\bar{W}}_{m}$ in order to maximize *V_trough_*. This feature can be seen from the distinct shift of the optimal *v_fiber_* from 3.5 mm/s to 4.1 mm/s under SD = 0.25 mm, and from 0.5 mm/s to 3.5 mm/s under SD = 0.50 mm, when the bubble collapse toward the stone surface was eliminated. This observation is consistent with previous studies, in which a faster scanning speed (e.g., 3 mm/s vs. 1 mm/s) was found to produce higher dusting efficiency regardless of F in contact mode [13]. Under similar settings (0.5 J, 20 Hz) using another laser lithotripter with different pulse profiles, the maximum dusting efficiency could be achieved at an even higher *v_fiber_* of 10 mm/s [23]. However, since the fiber speed and SD are difficult to control precisely during clinical LL, it is worth noting that a slow *v_fiber_* is much easier to manipulate and preferable over fast scanning speeds to avoid unintentional ureteral wall injury [23].

As a case study, the clinical implication of our results can be further illustrated by examining the optimal F for a given *v_fiber_* under the laser settings used in this work. Physically, the characteristics in the stone damage trough produced by a laser are primarily determined by the OAR (Figure 7), which unifies *v_fiber_* and F into a ratio correlating with the inter-pulse distance, as shown in Equation (2). As such, the same OAR between two successive laser pulses during scanning will ensure similarities in laser–fluid–bubble–stone interaction and resultant dusting damage, independent of the specific values of *v_fiber_* and F used. Consequently, we may estimate the optimal F under clinically relevant *v_fiber_* within the range of 1 mm/s to 3 mm/s [13]. As an illustrative example, the results, summarized in Table 1 together with the procedure time, suggest that the optimal F for stone dusting in contact mode (SD = 0.10 mm) will vary from 6 Hz to 17 Hz, which is within the frequency range for conventional low-power Ho: YAG lasers [48]. In comparison, the optimal F for stone dusting using the non-contact mode (SD = 0.50 mm) will be much higher, increasing from 40 Hz to 120 Hz, which can only be produced by contemporary high-power/high frequency Ho: YAG lasers. Conversely, based on the correlation between the trough volume and OAR in Figure 7, one can estimate that operating a high frequency laser using the contact mode in the range of 40 Hz to 120 Hz will decrease the dusting efficiency by 56–66% at 1 mm/s, 33–61% at 2 mm/s, or 11–56% at 3 mm/s.

Furthermore, it is important to note that the procedure time correlates inversely with F. Using the respective optimal Fs, the procedure time for the non-contact mode will be significantly shorter (1/7) compared to its counterpart for the contact mode under clinically viable scanning speeds (1 to 3 mm/s). All in all, stone dusting via the non-contact mode at an optimal SD (e.g., 0.50 mm) will allow for maximal cavitation damage that offers competitive treatment efficiency per pulse achievable at much higher F and, thus, a greatly reduced procedure time, compared to the conventional photothermal ablation treatment strategy via the contact mode (e.g., SD = 0.10 mm).

Our study has several limitations that need to be addressed in the future. First, it should be noted that BegoStone phantoms used in this study have different optical properties than human kidney stones [46,49]. Future work using renal calculi of various chemical compositions and in a kidney-like tissue environment to account for the retropulsion effect are warranted to determine the optimal stone dusting strategy in vivo. Second, the laser used in this study has relatively low F (<25 Hz). Additional experiments using high-frequency and high-power lasers may help to validate the correlation of *v_fiber_*, F, and SD with dusting efficiency. Third, stone damage mechanisms (i.e., photothermal vs. cavitation) may vary with laser pulse duration, shape, and modulation sequences [2,25,46]. Thus, a comprehensive study is warranted to determine the optimal dusting settings for different pulse profiles and sequences.

## 5. Conclusions

In this study, we have investigated the effects of fiber scanning speed and SDs on stone dusting during short pulse Ho: YAG LL. Our results suggest that dusting efficiency during scanning treatment can be significantly improved by the optimal combination of *v_fiber_*, F, and SD. Moreover, the substantial reduction in stone damage by mitigating bubble collapse toward the stone surface via the scope proximity clearly demonstrates the indispensable role of cavitation in stone dusting at various SDs during short pulse Ho: YAG LL. Most importantly, compared to the conventional photothermal ablation treatment strategy via the contact mode (e.g., SD = 0.10 mm), the non-contact mode at an optimal SD = 0.50 mm may offer a competitive treatment option for effective and efficient stone dusting leveraged by maximizing cavitation damage under high F with a shortened procedure time.

## Figures and Tables

**Table 1:** Comparison of the optimal pulse repetition frequency (F) and procedure time (t) under three clinically relevant fiber speeds (*v_fiber_*) as follows 1 mm/s, 2 mm/s, and 3 mm/s at a fiber-to-stone standoff distance (SD) of 0.10 mm (contact mode) and 0.50 mm (non-contact mode).

**SD = 0.10 mm (Contact Mode)**			
Optimal $v_{fiber}$ at F = 20 Hz	Optimal F at Different $v_{fiber}$		
	1 mm/s	2 mm/s	3 mm/s
$3.5\;\mathrm{mm}/s\;(\frac{v_{fiber}}{F}=0.175)$	6 Hz	11 Hz	17 Hz
Time required for 100 pulses, t_contact_	17.5 s	8.7 s	5.8 s
**SD = 0.50 mm (non-contact mode)**			
$0.5\;\mathrm{mm}/s\;(\frac{v_{fiber}}{F}=0.025)$	40 Hz	80 Hz	120 Hz
Time required for 100 pulses, t_non-contact_	2.5 s	1.3 s	0.8 s
Ratio of treatment time, t_contact_:t_non-contact_	7:1		

## Data Availability

Data are available by contacting authors.

## Supplementary Materials

The following supporting information can be downloaded at: https://www.mdpi.com/article/10.3390/jcm11175048/s1, Video S1: Bubble dynamics produced at SD = 0.25 mm and *v_fiber_* = 3.5 mm/s by the 20th pulse in water.
